# Biological Functions of Exosomes in the Liver in Health and Disease

**DOI:** 10.5812/hepatmon.13514

**Published:** 2014-05-10

**Authors:** Abbas Ali Imani Fooladi, Hamideh Mahmoodzadeh Hosseini

**Affiliations:** 1 Applied Microbiology Research Center, Baqiyatallah University of Medical Sciences, Tehran, IR Iran

**Keywords:** Exosomes, Liver Disease, Homeostasis, Drug Metabolism

**Figure 1.**
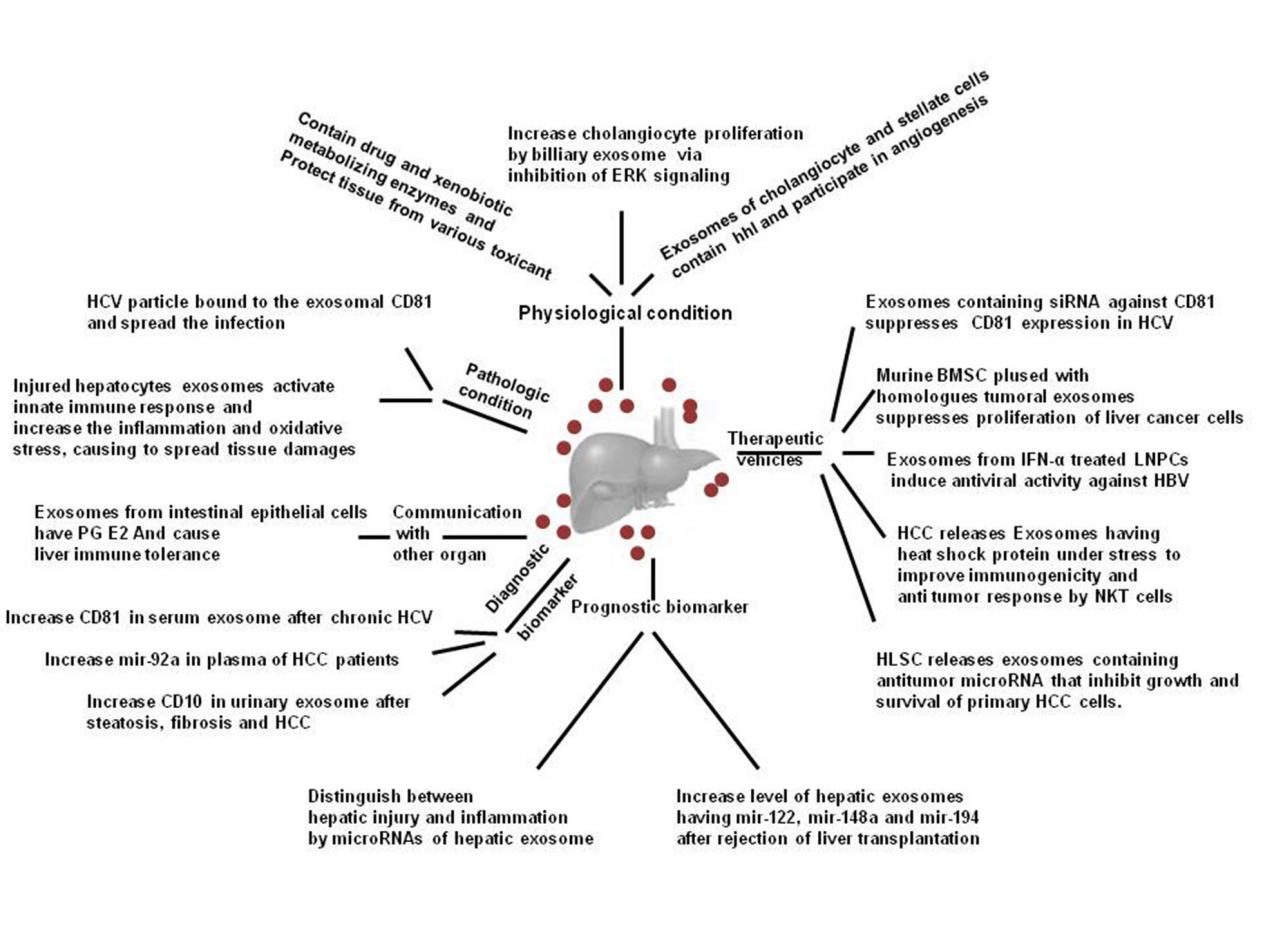
Schematic Illustration of Roles of Exosomes Derived From Liver Cells in Physiological and Pathological Condition, as Prognostic as Well as Diagnostic Biomarker, and as the Future Therapeutic Vehicles (Abbreviations: Exosomes; hhl, hedgehog ligand; HCV, hepatitis C virus; PG E2, prostaglandin E2; HCC, hepatocellular carcinoma; BMSC, bone marrow stromal cells; LNPCs, liver non parenchymal cells; HB, hepatitis B virus; NKT, natural killer cells; and HLSC, human adult liver stem cells).

The liver is a complicated environment comprised of several different cell types such as hepatocytes, parenchymal cells, Kupffer cells, natural killer cells, B cells, T cells, and stellate cells (1, 2), which are involved in various functions necessary for maintaining of the health. To maintain the proper function of the liver, coordination between all cell types population is necessary. One of the potent mechanisms involved in this harmonic orchestra is the exosome, 30-100 nm extracellular nanovesicles with the endosomal origin. Exosome contains various proteins, lipids, and genetic materials including mRNAs and microRNAs (miRNAs), which is closely associated with the content of cell released from. After secretion, the exosome can attach to the target cell, fuse with the membrane, deplete the cargos and finally, it can exchange the biological information with recipient cells that leads to promoting or inhibiting diverse signals. Hosseini et al. have published a useful review to shed light on these subjects (3). In spite of few data concerning the role of secreted exosome by liver cells, it is approved that the type and content of exosomes are different in the physiological and pathological conditions. This discrepancy can conduct the fate and behavior of the cells (Figure 1) (4). For instance, in the oxidative stress, released exosome have a transcriptome to modulate stress conditions via altering a pattern of gene expression. This event leads to prime the recipient cells for combating the new condition.

The presence of the drug and xenobiotic metabolizing enzymes such as cytochrome p450 family, uridine nucleotide diphosphate glucuronosyltransferase (UDPGT) and glutathione S- transferase within the hepatic exosomes and their release in human plasma has been confirmed (5). Therefore, these vesicles can assist tissues other than the liver to protect themselves against various toxicant. In addition, vesicle secretion from the cell may play a significant role in the emerging drug resistance. Hennessy et al. revealed that the presence of P-glycoprotein, which is involved in the multidrug resistance through the efflux of drug into the extracellular space, can be the cause of resistance in the sensitive cells after coculturing with the resistant cell lines (6). 

In addition to regenerating the injured tissues, exosome are involved in biological processes such as differentiation and angiogenesis. Exosomes released by the cholangiocytes and stellate cells contain the hedgehog ligands and participate in the vessels formation and angiogenesis (4). Furthermore, it is possible that the exosomes derived from cholangiocyte primary cilia participate in the detection and transduction of extracellular stimuli. Masyuk et al. revealed the positive effects of the biliary exosome on cholangiocytes proliferation via inhibition of ERK signaling (7). As described before, the exosomes are released in pathological conditions and contribute to promoting pathways involved in pathological processes such as inflammation, tumorigenesis, etc. In the cirrhotic and fibrotic liver, exosomes enriched from the shh, Ihh protein and the active hedgehog ligands are secreted by the cholangiocytes and stellate cells into plasma as well as bile. These exosomes are capable of modifying the gene expression patterns in the sinusoid endothelial cells and therefore, they may induce angiogenesis. One of the controversial aspect in the hepatitis C virus (HCV) infection is the assembly pathways of viral particles and their release into extracellular space. Previous observation demonstrated that the HCV particles and the viral RNA bound to the CD81 proteins. CD81 is a tetraspanin protein abundantly found in the exosome structure and is secreted from the infected cells. Since several membranous proteins consisting α4β1 integrin, ICAM (intercellular adhesion molecule), tetraspanin, and MHC (major histocompatibility complex) are represented on the surface of the exosome, this vesicle has a potency to attach to the vast majority of cells. Thus HCV particle bound to the exosomal CD81 might target the uninfected cells and consequently, spread the infection (4, 8). In addition to the hepatocytes, peripheral blood mononuclear cells (PBMCs) are capable of expressing CD81. Interaction of HCV particles with CD81 on the PBMCs leads to alleviate both the innate and adaptive immune response against HCV. This attenuating event leads to persistence of the infection. Data from Welker et al. demonstrated that the level of soluble CD81 is elevated in the chronic HCV infection and is associated with the hepatic inflammation and fibrosis (8). Nonalcoholic fatty liver disease (NAFLD) is the most prevalent liver disease worldwide. A broad spectrum of histological manifestations including macrovesicular steatosis, liver cirrhosis, fibrosis, portal hypertension, and hepatocellular carcinoma are observed in NAFLD. The exosomes released by injured hepatocytes include certain antigens, specific hepatic enzymes, miRNAs, and mRNAs that can trigger the activation of innate immune response, increase the inflammation in the liver and induce production of interleukins that leads to the oxidative stress and spreading tissue damages. Exosome released in this condition may release into blood and interact with the antigen presenting cells (APCs) located in the bone marrow and may promote an autoimmune response resulting in extensive liver injury (9). Bala et al. reported that miRNA presented in the exosome derived from the liver cells can distinguish between injuries and inflammatory conditions in the liver. They found that exosome secreted in the inflammatory situations such as alcoholic liver disease, has a significant amount of mir-155, whereas in the HCV infection and hepatocellular carcinoma, mir-122 enriched exosomes are abundant (10). To our knowledge, exosomes contribute in both the liver restoration and damaging; therefore controlling their release may be useful for prevention and treatment of liver diseases. In addition, designing a novel tool based on exosome to apply in gene therapy may be useful.
